# Mesenchymal Stromal Cells Prevent Renal Fibrosis in a Rat Model of Unilateral Ureteral Obstruction by Suppressing the Renin-Angiotensin System via HuR

**DOI:** 10.1371/journal.pone.0148542

**Published:** 2016-02-11

**Authors:** Marilena Gregorini, Valeria Corradetti, Chiara Rocca, Eleonora Francesca Pattonieri, Teresa Valsania, Samantha Milanesi, Nicoletta Serpieri, Giulia Bedino, Pasquale Esposito, Carmelo Libetta, Maria Antonietta Avanzini, Melissa Mantelli, Daniela Ingo, Sabrina Peressini, Riccardo Albertini, Antonio Dal Canton, Teresa Rampino

**Affiliations:** 1 Unit of Nephrology, Dialysis, Transplantation, Fondazione IRCCS Policlinico San Matteo and University of Pavia, Pavia, Italy; 2 Laboratory of Transplant Immunology/Cell Factory Fondazione IRCCS Policlinico S. Matteo, Pavia, Italy; 3 Clinical Chemistry Laboratory Fondazione IRCCS Policlinico San Matteo, Pavia, Italy; Center for Molecular Biotechnology, ITALY

## Abstract

We studied Mesenchymal Stromal Cells (MSC) effects in experimental Unilateral Ureteral Obstruction (UUO), a fibrogenic renal disease. Rats were divided in 5 groups: sham, UUO, MSC treated-UUO, ACEi treated-UUO, MSC+ACEi treated- UUO. Data were collected at 1, 7, 21 days. UUO induced monocyte renal infiltration, tubular cell apoptosis, tubular atrophy, interstitial fibrosis and overexpression of TGFβ, Renin mRNA (RENmRNA), increase of Renin, Angiotensin II (AII) and aldosterone serum levels. Both lisinopril (ACEi) and MSC treatment prevented monocyte infiltration, reduced tubular cell apoptosis, renal fibrosis and TGFβ expression. Combined therapy provided a further suppression of monocyte infiltration and tubular injury. Lisinopril alone caused a rebound activation of Renin-Angiotensin System (RAS), while MSC suppressed RENmRNA and Renin synthesis and induced a decrease of AII and aldosterone serum levels. Furthermore, in *in-vitro* and *in-vivo* experiments, MSC inhibit Human antigen R (HuR) trascription, an enhancer of RENmRNA stability by IL10 release. In conclusion, we demonstrate that in UUO MSC prevent fibrosis, by decreasing HuR-dependent RENmRNA stability. Our findings give a clue to understand the molecular mechanism through which MSC may prevent fibrosis in a wide and heterogeneous number of diseases that share RAS activation as common upstream pathogenic mechanism.

## Introduction

Renal fibrosis represents the common ending of several renal diseases. Experimental Unilateral Ureteral Obstruction (UUO) has provided extensive information on renal fibrogenesis. [1–3] Different studies have shown that UUO causes a sustained glomerular ischemia, that is associated with the activation of the Renin Angiotensin System (RAS). [4–6] Angiotensin II (AII) stimulates the production of nuclear NF-kB, which recruits macrophages that release reactive oxygen species [7,8] and Tumor Necrosis Factor α (TNFα), an inducer of apoptosis and tubular atrophy. [1,9] Moreover, inflammatory cells release Transforming Growth Factor β (TGFβ) that promotes epithelial mesenchymal transition of tubular epithelial cells, while fibroblasts synthesize stress fibres and deposit extracellular matrix causing progressive interstitial fibrosis.[10–13] Furthermore, recent studies give evidence that Renin binding with its (pro)renin receptor directly prompts the extracellular-signal-regulated kinase (ERK)-dependent production of TGFβ. Therefore, Renin has a fibrogenic effect that is independent of its enzymatic action on angiotensinogen.[14–16] Human antigen R (HuR) has been recently described to stabilize RENmRNA sustaining Renin production. In the same time it has been demonstrated that HuR expression was inhibited by IL10.[17–19] Currently, RAS is the only fibrogenic system targeted by drug therapy. In fact angiotensin-converting enzyme inhibitors (ACEi) and AII receptor blockers are used in clinic to slow down the progression of renal diseases.[20–27] MSC are isolated from several human tissues, can differentiate in multiple adult cell lineages, and display immunomodulatory properties.[28–30] When injected in animal disease models MSC migrate to sites of inflammation and injury and secrete paracrine mediators for tissue repair.[31–36] These pleiotropic activities have prompted the use of MSC for treatment of diseases where cell damage is accompanied by local and systemic inflammatory response [37–40] and for prevention of fibrosis in animal models and clinical studies.[41–44]

Here we show that MSC prevent fibrogenic kidney injury in experimental UUO and compare their effects with those of lisinopril, an ACEi. Furthermore, we report on the potential therapeutic advantage of their combined use. Finally we show that MSC suppress RAS, inhibiting HuR by IL10 release.

## Materials and Methods

### 2.1 Animals

61 male Wild-type Sprague Dawley (SD) rats (Charles River, Lecco, Italy) weighed 250–300 g, were used for animal model. While 6 transgenic Enhaced Green Fluorescent Protein (EGFP) SD rats (Japan Slc, Hamamatsu, Japan) were used to expand MSC.[45]

Rats were housed at constant temperature (20 C°) and humidity (75%) under controlled light cycle, with free access to water and standard chow diet.

### 2.2 Isolation and Colture of MSC

Transgenic EGFP SD rats were sacrificed by CO_2_ inhalation. Marrow cells were flushed from femurs and tibias and mononuclear cells were isolated by density gradient centrifugation (Ficoll 1077 g/ml; Lympholyte, Cedarlane Laboratories Ltd.) counted and plated in 75 or 175 cm^2^ tissue culture flasks at a density of 160000/cm² in αMEM supplemented with 10% murin MesenCult (Voden, Milan, Italy) and 1% antibiotic-antimycotic (Sigma-Aldrich, Saint Louis, US). Cultures were maintained at 37°C, 5% CO_2_ in a humidified atmosphere. After 48 hours, non-adherent cells were removed and culture medium was replaced twice a week. After reaching ≥80% confluence, MSC were harvested using Trypsin-EDTA (Lonza, Copenhagen, Denmark), and propagated at 4000 cells/cm².

MSC were expanded until passage (P) 4. At each passage, viable cells were counted using 0.1% eosin and culture supernatants were tested for sterility.

### 2.3 Immunophenotype Characterization

EGFP-MSC were characterized by flow-cytometry. Phycoerythrin (PE)-conjugated monoclonal antibodies (BD PharMingen, San Diego, US) against rat CD11b, CD45, CD90, CD49e and CD29 (Becton Dickinson, Franklin Lakes, US) using FacsCalibur flow cytometer (BD Biosciences) were used as described.[41] Appropriate, isotype-matched, non-reactive fluorochrome-conjugated antibodies were used as controls.

### 2.4 Differentiation assays

EGFP-MSC were evaluated for their ability to differentiate into osteoblasts and adipocytes, as previously described.[28]

The osteogenic differentiation capacity of MSC was assessed at P2-4 by incubating cells with αMEM, 10% FBS, 1% gentamicin, supplemented with 10^-7^M dexamethasone (Sigma-Aldrich, St Louis, US), 50 mg/ml L-ascorbic acid (Sigma-Aldrich). Starting from day 7 of culture, 5 mM β-glycerol phosphate (Sigma-Aldrich) was added to the medium. Adipogenic differentiation was evaluated at P2-4 by incubating cells with αMEM, 10% FBS, 1% gentamicin supplemented with 10^-7^M dexamethasone, 50 mg/ml L-ascorbic acid, 100 mg/ml insulin, 50 mM isobutyl methylxanthine (Sigma-Aldrich), 0.5 mM indomethacin (MP Biomedica, Illkirch, France) and 5 mM β-glycerol phosphate. Both osteogenic and adipogenic cultures were incubated for 21 days before evaluating differentiation. To detect osteogenic differentiation, cells were stained for alkaline phosphatase activity using Fast Blue (Sigma-Aldrich) and, for calcium deposition, by Alizarin Red S staining (Sigma-Aldrich). Adipogenic differentiation was evaluated through the morphological appearance of fat droplets stained with Oil Red O (Bio Optica, Milan, Italy).

### 2.5 *In vivo* experiments

In vivo experiments were performed according to guidelines of our ethical committee for animal studies and approved by ministerial ordinance n° 36262.

SD rats were anesthetized using intramuscular injection of Zolazepam-Tiletamine (30 mg/Kg) (Zoletil). After the induction of anesthesia a flank incision was made and the right ureter was ligated with 5/0 silk suture to induce obstruction. Animals were divided in 5 groups: Sham: 8 rats sham operated. Vehicle: 8 rats received 1 ml of saline solution after UUO at day 0 via tail vein. MSC: 8 rats received one dose of MSC (3X10^6^) after UUO at day 0 via tail vein. ACEi: 8 rats received lisinopril in the drinking water (100 mg/L) from days 1 to 21. ACEi+MSC: 8 rats received one dose of MSC (3X10^6^) on day 0, and ACEi from days 1 to 21. Rats were sacrificed at day 7 and 21 after ureteral ligation. Blood samples were collected at 7 and 21 days, centrifuged and maintained at -20°C until analyses were performed.

Another set of experiments was performed using 3 groups of rats sacrificed 1 day after ureteral ligation: Sham: n = 5, Vehicle: n = 8, MSC: n = 8. Soon after sacrifice kidneys were removed, half was fixed in 10% neutral-buffered formalin, half frozen in liquid nitrogen.

### 2.6 Biochemical measurements

Serum creatinine levels were measured by an automated method (Abbott Laboratories, Chicago, Illinois, US). Serum AII levels were quantified by ELISA (LifeSpan BioSciences, Newton, US). Serum aldosterone levels were measured by chemiluminiscent assay (Liaison Aldosterone assay, DiaSorin, Vercelli, Italy).

For dosage of Renin and TGFβ in renal tissue the kidneys were homogenized in 0.1 M tris HCL, pH 7.4, containing 8-hydroxyquinolone sulfate (3.4mM), 0.25 EDTA, 0.1 phenylmethysulfonyl fluotide, 1,6 dimercaprol, 5 sodium tetrathionate, and 0.1% Triton X-100. The concentration of proteins was determined with a bicinchoninic acid protein assay kit (Pierce, Rockford, IL, US). After centrifugation of the homogenate, supernatant was collected and stored at -70°C. Renin levels were measured in renal tissue at day 7 by chemiluminiscent assay (DiaSorin), using monoclonal antibody (mouse) that recognizes Renin and prorenin. TGFβ levels were measured in renal tissue by Quantikine ELISA Kit (R&D Systems, Minneapolis, US), that recognizes rat TGFβ in surnatant.

### 2.7 RNA Extraction and Real time PCR

Total RNA was extracted from renal tissue and cultured cells (HK2 cell line and macrophages) using respectively trizol method and guanidine based RNeasy^®^ Mini Kit (QIAGEN Venlo, Holland). RNA was treated with DNase from RNase-Free DNase Set (QIAGEN) and dissolved in nuclease free water. Extracted RNA was tested for quantity and integrity by spectrophotometric analysis (NanoDrop, Thermo Scientific, Watham, US). A total of 1 μg of RNA per condition was reverse transcribed into cDNA through 1st Strand cDNA Synthesis Kit for RT-PCR (AMV) (Roche Applied Science, Basel, Germany). cDNA was used to perform real-time PCR analysis in 96-well optical reaction plates, using ABI prism 5700 (Applied Biosystems, Watham, US) and the 5-exonuclease assay (TaqMan technology) in a volume of 25 μl reaction containing Taqman Universal Master Mix, optimized concentrations of FAM-labeled probe, and specific forward and reverse primers for βactin, Renin and HuR (Applied Biosystems, Watham, US; catalogue number Rn00667869_m1, Rn00561847_m1 and Rn01403240_m1, respectively). Water replacing cDNA was included in real-time PCR as controls. The results were analyzed using a comparative method, and values normalized to the βactin expression and converted into fold change.

### 2.8 Renal Histopathology

#### 2.8.1 Apoptotic nuclei

Renal sections were dewaxed in xylol, passed in a decreasing series of alcohol, and finally rehydrated with distilled water. For permeabilization the tissues sections were exposed to distilled water containing 0.1%Triton X-100, 0.1% citrate sodium, then incubated with proteinase K in Tris/HCl10mM, pH 7.4–7.8. After washing in PBS, sections were incubated with 20 ml of TUNEL reaction mixture (In Situ Cell Death Detection Kit, Fluorescein, Roche) for 1 hour at 37°C, in a dark humid chamber. After washing in PBS, specimens were mounted in Vectashield Mounting Medium with DAPI (Vector Laboratories, Burlingame, US) and examined with fluorescence microscope Nikon E400 with CCD camera and software imaging analysis Cell-R. In order to assess apoptotic nuclei we examined 10 non-overlapping fields per renal section (X400) per animal and expressed them as means ± SD.

#### 2.8.2 Renal Morphology

3 μm thick sections of formalin-fixed kidney were examined by two investigators in double blind fashion, using an Olympus IX8 microscope connected with a CCD camera and software imaging analysis Cell-R.

Tubule diameters were analyzed in 10 non consecutive fields (X400) per kidney section. Results were expressed as μm (means ± SD). The percentage of broken tubules/High Power Field (HPF) was evaluated in 10 non consecutive fields (X400) per renal section. Results were expressed as percentage of broken tubules/HPF. Interstitial fibrosis was measured by Masson’s trichrome staining (score 1–4): 1: Masson’s trichrome positive area/HPF <25%, 2: Masson’s trichrome staining involving 26 to 50%, 3: Masson’s trichrome staining involving 51 to 75%, 4: Masson’s trichrome staining involving >75%. The results were expressed as means ± SD.

#### 2.8.3 EGFP, ED1 Ag and Fibroblast Specific Protein 1 (FSP1) expression

EGFP, ED1 Ag and FSP1 renal expression were studied by immunohistochemistry (IHC) in formalin fixed tissue. Three-μm thick sections of paraffin embedded tissue were collected on poly-L-lysine-coated slides (Dako, Glostrup, Denmark), they were dewaxed in xylol, passed in a decreasing series of alcohol, and finally rehydrated with distilled water. Endogenous peroxidase was blocked with H_2_O_2_/methanol 3.7% vol/vol for 10 minutes followed by H_2_O. After 3 washings in PBS sections underwent microwave antigen retrieval, then were exposed overnight at 4°C to the following antibodies: 1) monoclonal mouse anti-green fluorescent protein antibody IgG1 (Chemicon International, Billerica, US), 2) monoclonal mouse anti-rat ED1 (Serotec Ltd, Kidlington, UK), 3) polyclonal anti S100-A4 (Dako). After 3 washings in PBS the immunocomplex was visualized with the biotin-streptavidin-peroxidase complex and 3,3-diaminobenzidine (Dako). Sections were faintly counterstained with Harris hematoxylin. Negative controls included both omission of the primary Ab and substitution of IgG for primary antibodies. The positive controls for EGFP were kidney sections of SD-EGFP. In 10 renal sections per kidney we counted EGFP, ED1 and FSP1 positive cells/HPF. Since EGFP cells were spontaneously fluorescent we studied EGFP renal expression also by fluorescence method. Renal sections were air dried and post-fixed in ethanol 70%, 95%, 100% (3 min). After washing in PBS, specimens were mounted in Mowiol Mounting Medium (Sigma-Aldrich) and examined with fluorescence microscope Nikon E400 with CCD camera and software imaging analysis Cell-R.

### 2.9 *In vitro* experiments

HK2 cell line (ATCC^®^ CRL-2190^™^, Manassas, US) was used to assess Renin production in co-culture experiments. Cells were planted in 6-well plates at concentration of 1x10^6^/well in Low Glucose (LG) Medium: RPMI 1640, 10% Fetal Calf Serum (FCS) (EuroClone, Pero, Italy), 1% insulin-transferrin-selenium and 1% penicillin/strepromycin (Gibco, Waltham, UK), and incubated at 37°C, 5% CO_2_. High Glucose (HG) medium was prepared adding 6 g/L (30mM) of glucose to LG medium. MSC/HK2 were planted in well at ratio of 1:2, 1:20 and 1:200 in LG medium. After 24h, medium was replaced with HG-medium for 4h then cells were collected for RNA extraction.

In additional experiments, HK2 were cultured in HG: a) additioned with IL10 (10 ng/ml) (R&D Systems) for 1h, b) co-cultured with MSC (1:2) and anti IL10 blocking antibody (100 ng/ml) (Pierce-Endogen, Rockford, US) for 4h. Cells were collected for RNA extraction.

Monocytes (MC) were isolated from heparinized blood by density gradient centrifugation (Lymphoprep, Stemcell Technologies, Vancouver, Canada), and plated at 1x10^6^/well in RPMI1640 supplemented with 10% human AB positive serum, 1% penicillin/streptomycin to induce macrophage (Mϕ) differentation. After 24h of adherence cells were scraped. Mϕ differentiation was confirmed by flow cytometer analysis using anti CD14, anti CD11b, anti CD31 and anti CD62L (BD Pharmingen). Mϕ were than cultured with MSC at different ratios (MSC/Mϕ 1:2, 1:20 and 1:200) for 4 days at 37°C, 5% CO_2_.

In additional experiments, Mϕ were cultured: a) with IL10 (10 ng/ml) (R&D Systems) for 4h, b) co-cultured with MSC (1:2) and anti IL10 blocking antibody (100 ng/ml) (Pierce-Endogen) for 24h. Cells were collected for RNA extraction.

### 2.10 Statistical analysis

ANOVA followed by the Newman-Keuls test were used to compare not parametric variables and Student t test for the means.

## Results

### 3.1 Rat MSC characterization

**Fig 1:**
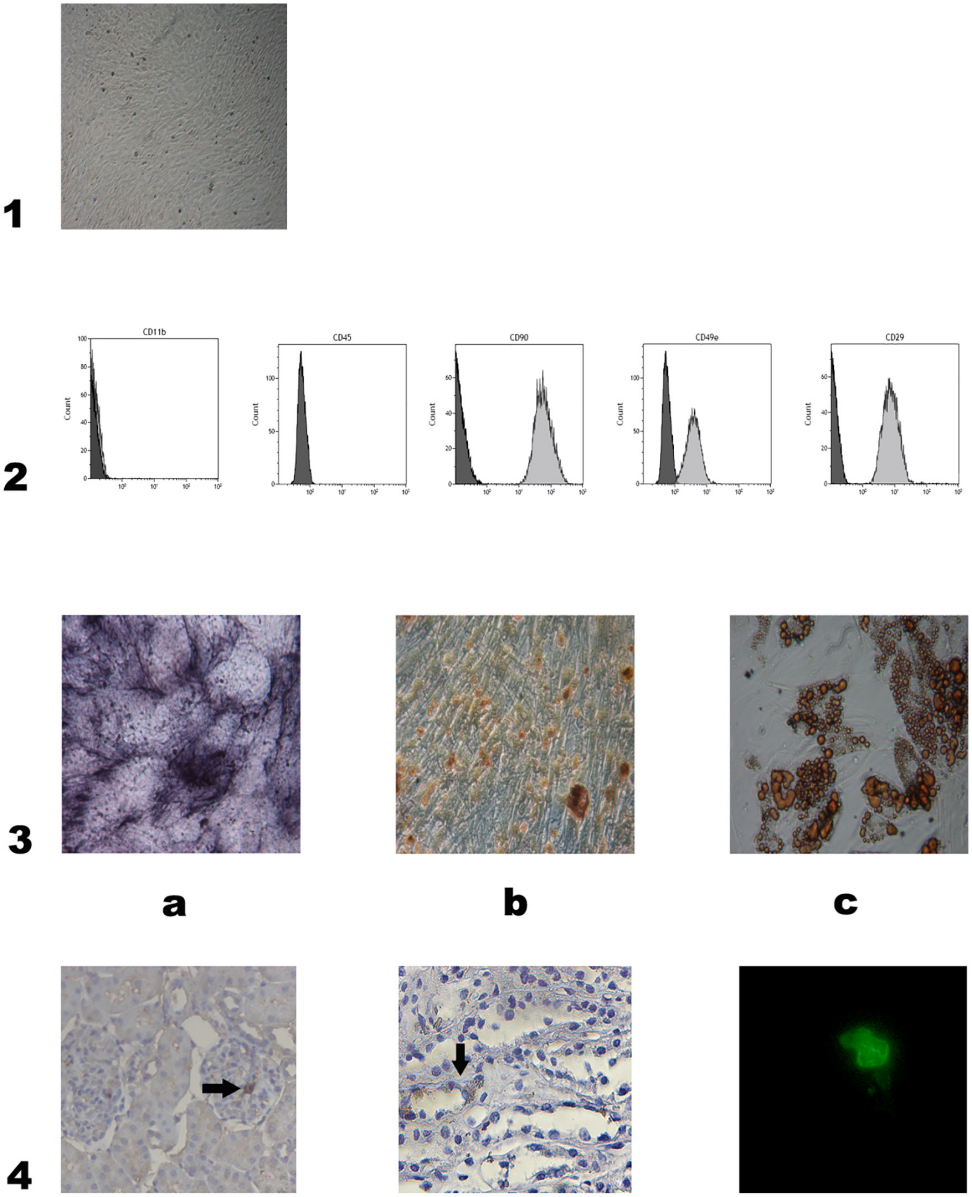
Characterization and localization of rat EGFP-MSC. 1: Characteristic spindle-shaped morphology of bone marrow derived MSC obtained from one rat. Magnification X4. 2: Immunophenotype of one representative culture-expanded rat EGFP-MSC. Rat MSC were negative for CD11b and CD45 and positive for CD90, CD49e and CD29. 3: Osteogenic and adipogenic differentiation capacity of rat MSC. Differentiation into osteoblasts was demonstrated by the histological detection of Alkaline phosphatase activity (a) and calcium depositions positive for Alizarin Red (b). Differentiation into adipocytes was revealed by the formation of lipid droplets stained with Oil Red O (c). Magnification X20. 4: Representative renal sections of rats treated with MSC sacrified one day after ureteral ligation. Arrows indicate EGFP positive MSC. Immunohistochemistry for EGFP showing glomerular (a) tubular and interstitial (b) localization of MSC, magnification X200. Self fluorescence showed EGFP-MSC presence (c), magnification X400.

EGFP-MSC showed the typical spindle shape morphology (Fig 1, panel 1) and resulted positive for CD49, CD90, and CD29, while they were negative for CD45 and CD11b. (Fig 1, panel 2) MSC exhibited the capacity to differentiate *in vitro* to osteoblasts and adipocytes as confirmed by histological staining. Microscopic examination of stained cells demonstrated the presence of mineralization nodules and fat droplets. (Fig 1, panel 3)

### 3.2 *In vivo* experiments

Through the study period, no significant differences were observed in body weight, water and food intake among the 5 groups of rats. No side effects were documented.

### 3.3 MSC localization

In EGFP-MSC treated rats, few cells were tracked (0–5 per section/HPF), on kidney sections obtained at day 1, and they were located in glomeruli, tubules and interstitium. (Fig 1, panel 4)

### 3.4 Renal function

**Fig 2:**
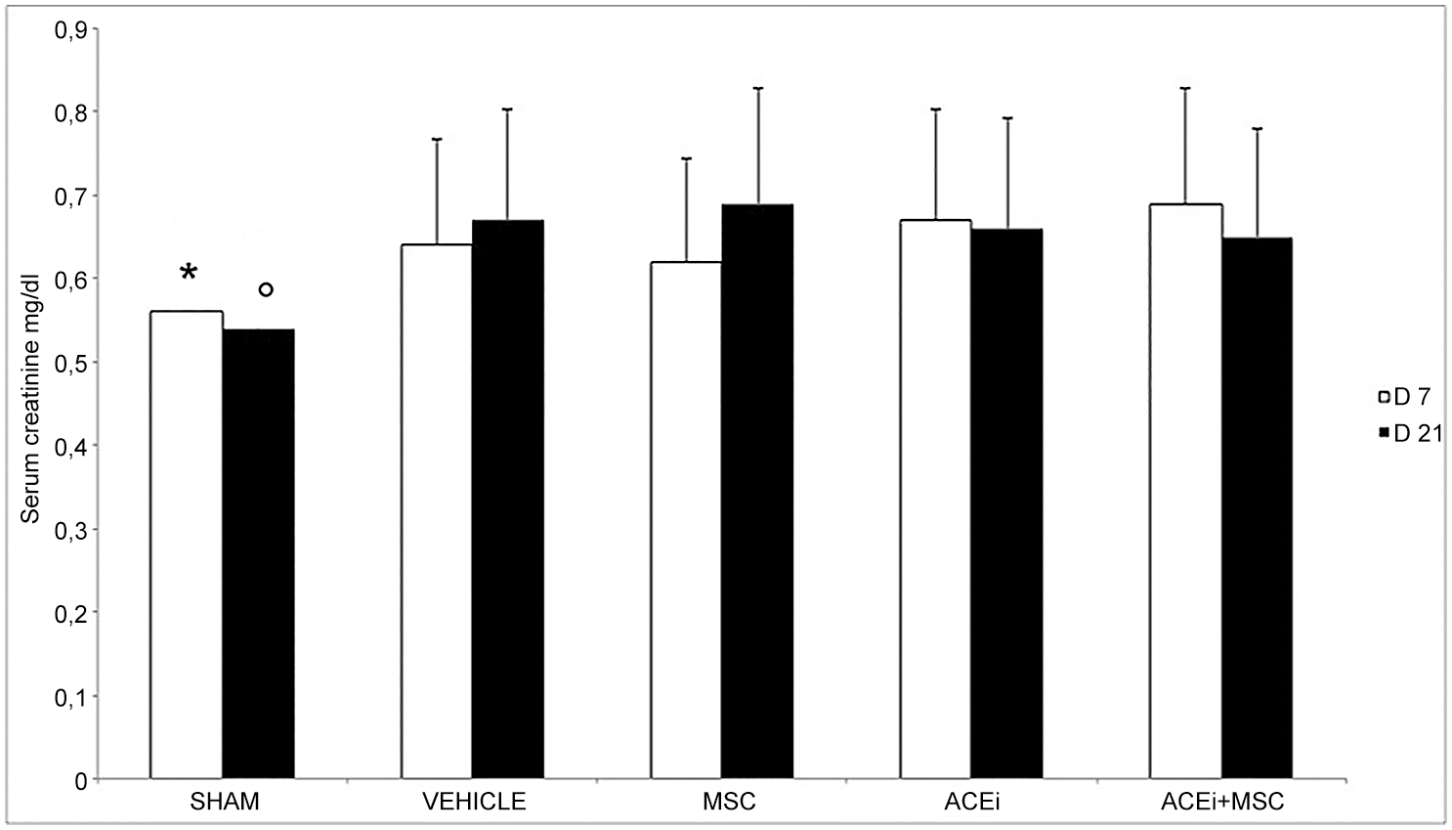
Serum creatinine levels at 7 and 21 days after UUO in sham, vehicle, MSC, ACEi, ACEi+MSC groups. D7 and D21 indicate the day after UUO, columns represent serum creatinine levels, bars represent SD. Data are mean ± SD. *p<0.05 vs all groups, °p<0.05 vs all groups.

At 7 and 21 days serum creatinine levels were significantly higher in UUO compared to sham (p<0.05). There was no difference among groups of obstructed rats, nor between day 7 and day 21 (Fig 2)

### 3.5 RENmRNA expression, Renin tissue levels, AII and aldosterone serum levels

**Fig 3:**
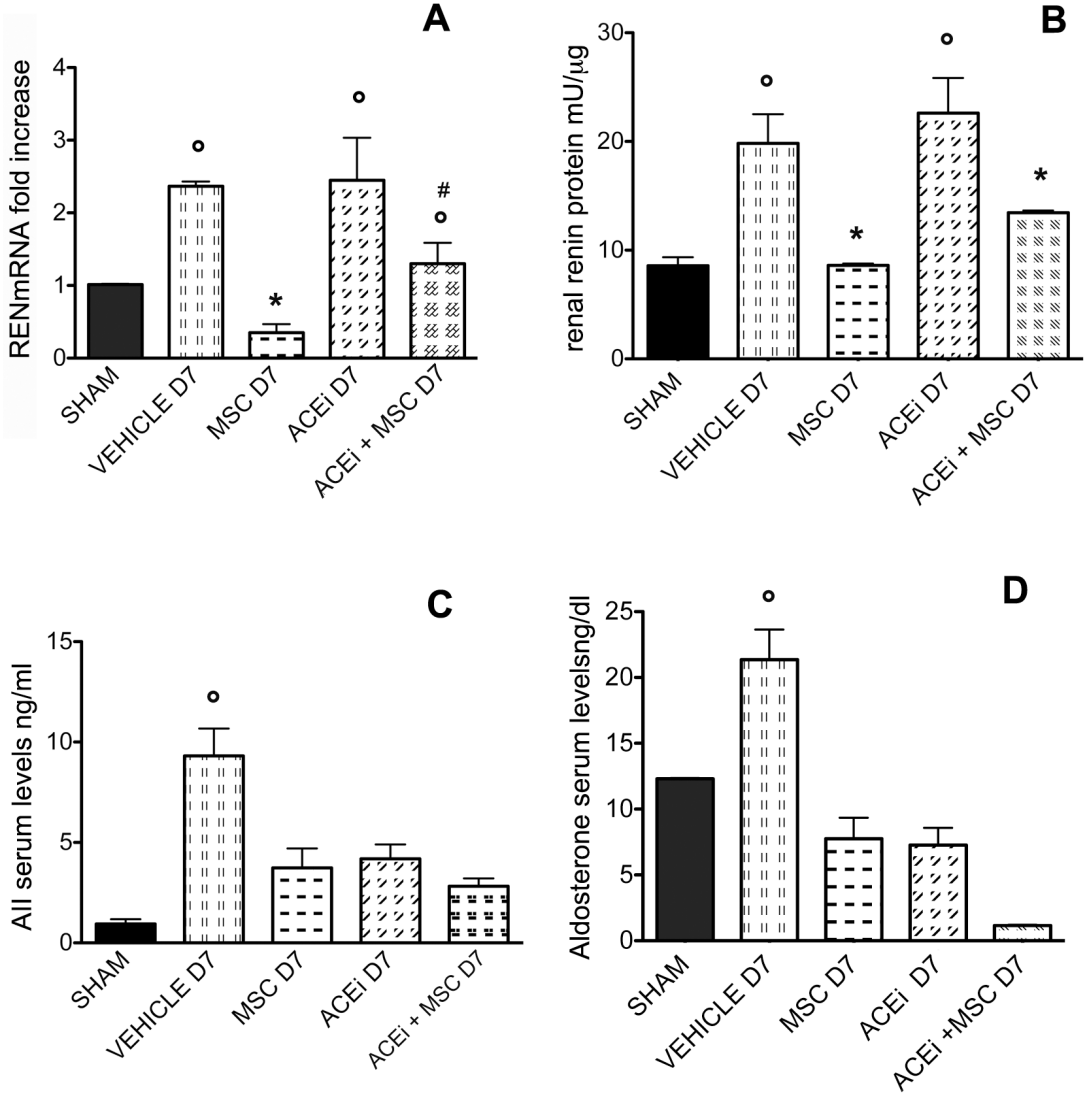
REN mRNA, renal Renin, serum AII and aldosterone levels. D7 indicates the day after ureteral ligation. A: RENmRNA expression in all groups of rats determined by real time PCR. Columns indicate RENmRNA expression normalized to the βactin expression and converted in fold change. °p<0.001 vs sham, *p<0.05 vs vehicle D7, ACEi D7 and ACEi+MSC D7, #p<0.05 vs ACEi D7. B: Renin protein levels in renal tissue in all groups of rats. Columns represent Renin protein levels. Data are means ± SD. °p<0.005 vs sham, MSC D7 and ACEi+MSC D7, *p<0.05 vs vehicle D7 and ACEiD7. C: AII serum levels in all groups of rats. Columns represent AII levels. Data are means ± SD. °p<0.005 vs all groups. D: Aldosterone serum levels in all groups of rats. Columns represent aldosterone serum levels. Data are means ± SD. °p<0.005 vs all groups.

After 7 days of UUO, RENmRNA expression significantly increased in renal tissue of all groups except for MSC D7 (p<0.001), while in MSC and ACEi+MSC groups RENmRNA expression decreased in comparison to vehicle and ACEi groups (p<0.05), suggesting a MSC inhibitory effect on RENmRNA expression. (Fig 3, panel A)

Accordingly, at day 7 Renin tissue levels increased in vehicle and ACEi rats compared to sham, while in MSC and ACEi+MSC groups they were significantly decreased compared to vehicle and ACEi rats (p<0.05). (Fig 3, panel B)

As sign of RAS activation, after 7 days AII and aldosterone serum levels significantly increased in UUO rats (p<0.005). MSC, ACEi and combined therapy induced a significant decrease in AII and aldosterone serum levels (p<0.005). (Fig 3 panels C and D, respectively)

### 3.6 Monocyte infiltrate

**Fig 4:**
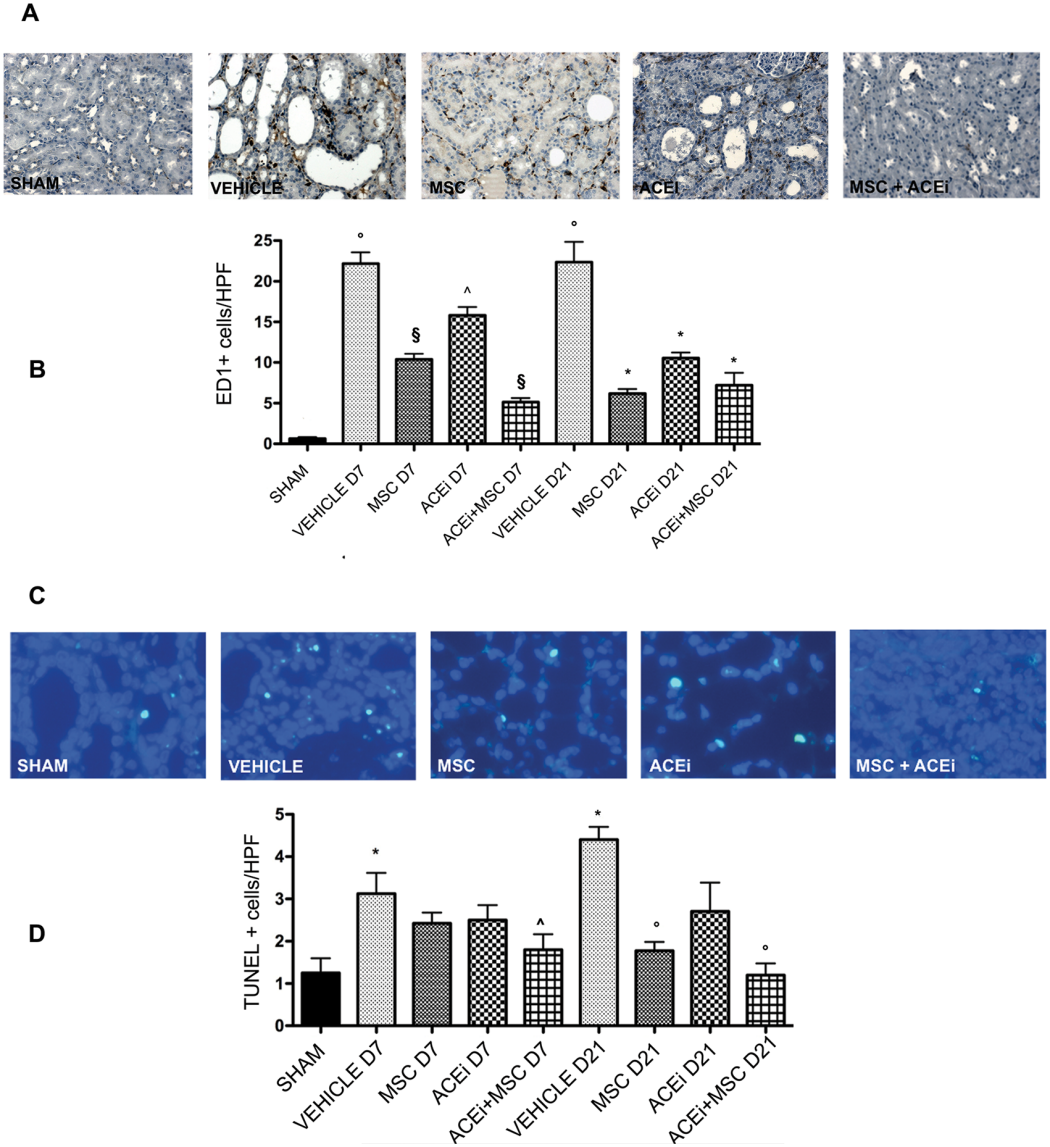
Monocyte infiltrate and tubular apoptosis. A: *Monocyte infiltrate*. Representative renal sections of all groups of rats sacrified 7 days after ureteral ligation stained for ED1 binding cells. Magnification, X200. B: Columns represent ED1 binding cells/HPF (means ± SD). Kidneys studied at day 7 and day 21. °p<0.0001 vs sham, §p<0.0001 vs vehicle D7, ^p<0.05 vs MSC D7 and ACEi+MSC D7, *p<0.0001 vs vehicle D21. C: *Tubular apoptosis*. Representative renal sections of all groups of rats sacrificed 21 days after ureteral ligation stained with DAPI to evaluate apoptotic nuclei. Magnification, X400. D: Columns represent apoptotic nuclei/HPF (means ± SD). Kidneys studied at day 7 and day 21. *p<0.05 vs Sham, ^p<0.05 vs Vehicle D7, °p<0.005 vs Vehicle D21.

As shown in Fig 4 panel B, UUO induced a significant MC infiltration increase in renal tissue (p<0.0001). After 7 days of ligation, MC were significantly reduced by MSC and ACEi+MSC (p<0.001). Moreover combined therapy significantly reduced MC infiltration compared to ACEi (p<0.05). At 21 day, MC infiltration was significantly reduced by all treatments (p<0.0001). (Fig 4, panel B)

### 3.7 Apoptosis

Tunel tubular positive cells increased significantly after 7 and 21 days of ureteral ligation in vehicle rats compared to sham operated rats (p<0.005). At day 7, ACEi+MSC treatment significantly decreased tubular cell apoptosis (p<0.05). At day 21 MSC and ACEi+MSC treatment significantly decreased tubular cell apoptosis (p<0.005). (Fig 4, panel D)

### 3.8 Tubular damage

**Fig 5:**
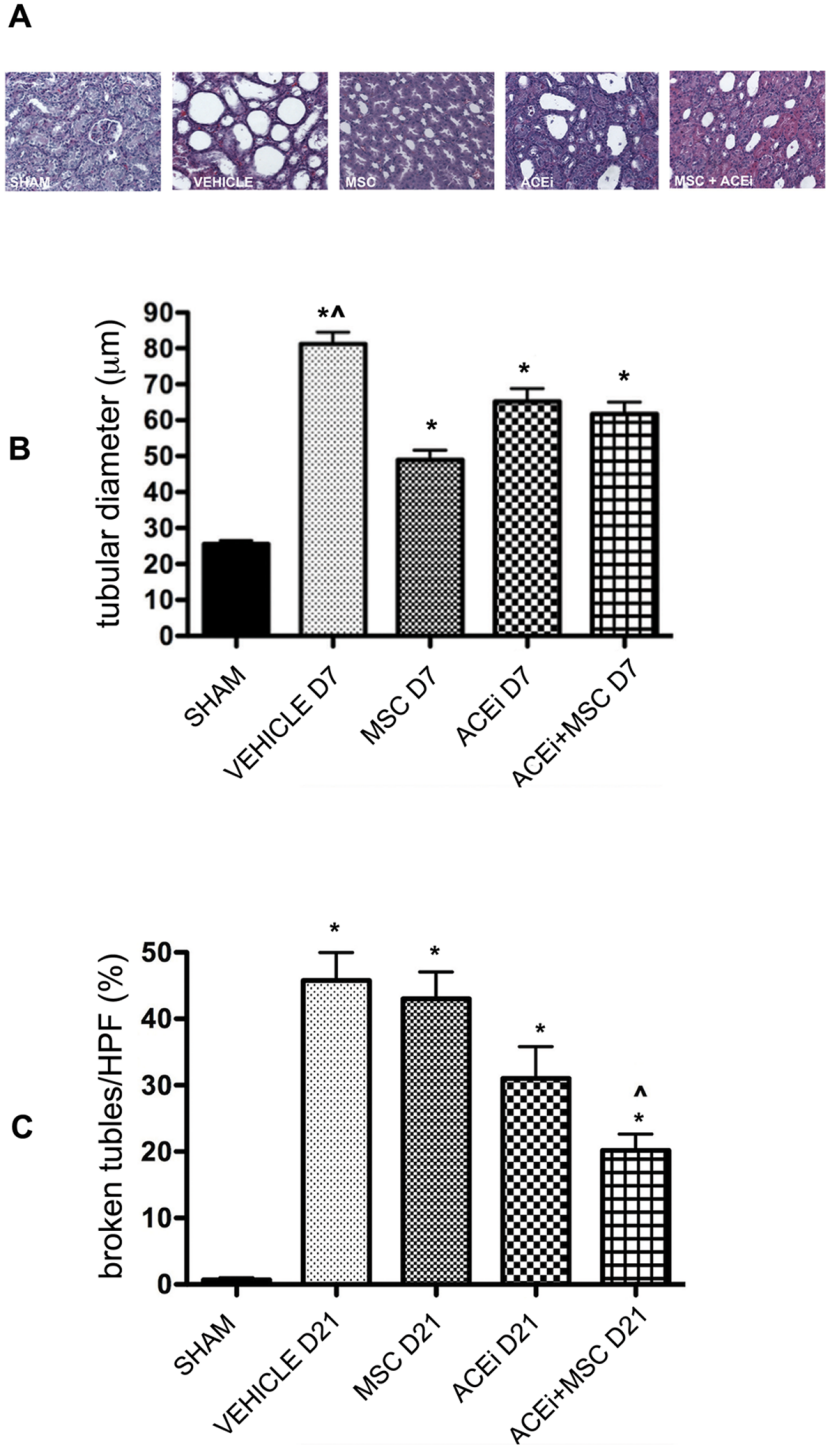
Tubular damage. A: *Tubular diameters*: Hematoxylin-eosin staining of representative renal sections of all groups of rats at day 7. Magnification, X200. B: *Tubular diameters* columns represent tubule diameters/HPF at day 7, results expressed as means ± SD. *p<0.0001 vs sham, ^p<0.05 vs MSC D7, ACEi D7 and ACEi+MSC D7. C: *Broken tubules*. Columns represent percentage of broken tubules/HPF at day 21. Results were expressed as means ± SD. *p<0.0001 vs sham, ^p<0.01 vs MSC D21 and Vehicle D21.

Tubular damage was evaluated at day 7 by measuring tubular diameters and at day 21 by enumerating damaged Tubular Basement Membrane (TBM). Tubular diameter was significantly increased in UUO compared to sham (p<0.0001). Renal sections of MSC, ACEi and ACEi+MSC treated rats showed tubules with significantly smaller diameters compared to vehicle (p<0.05). (Fig 5, panel B)

The number of tubules with damaged TBM was significantly reduced by combined therapy compared to vehicle and MSC alone (p<0.01). (Fig 5, panel C)

### 3.9 Renal tissue TGFβlevels

**Fig 6:**
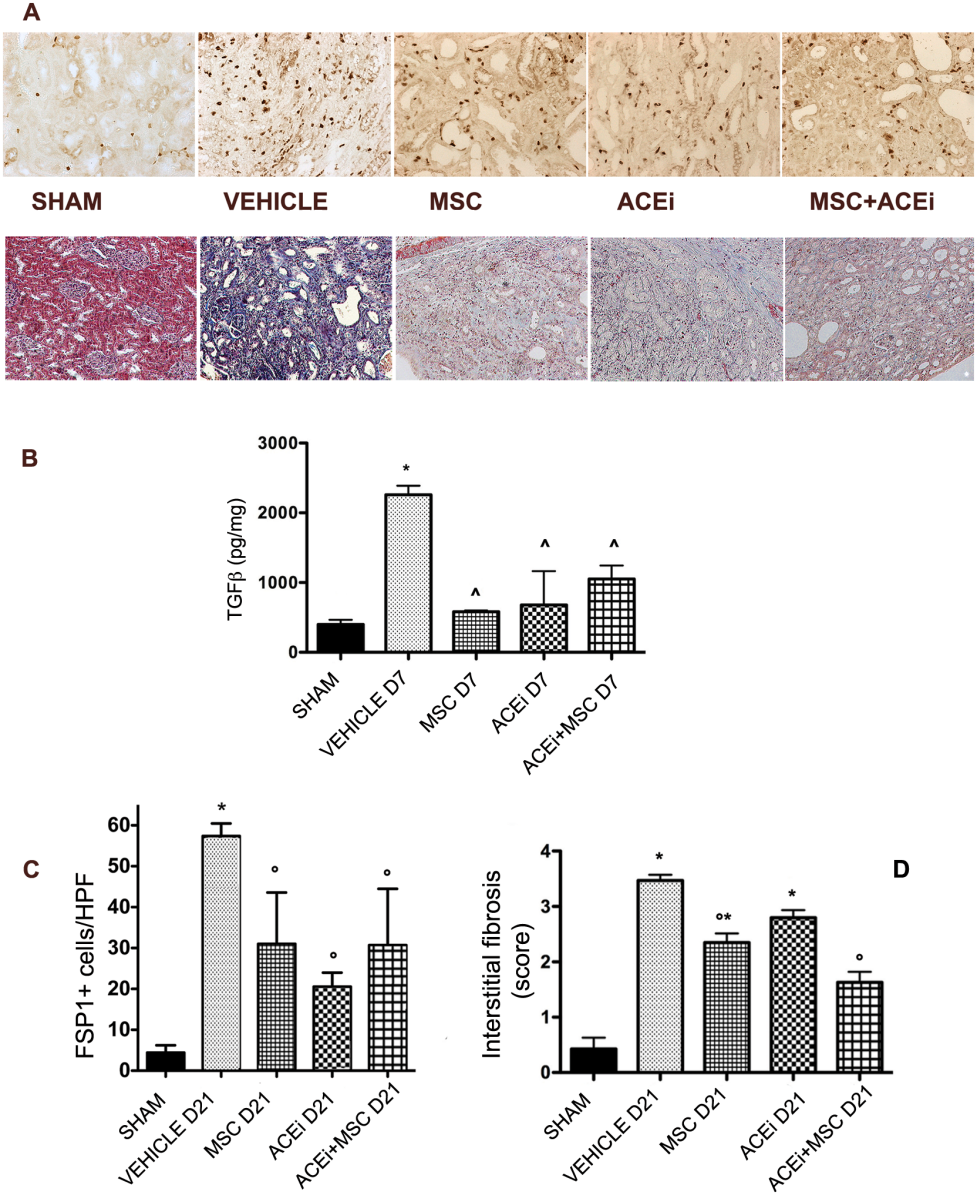
Renal fibrosis. A: Upper: Representative renal sections of IHC for FSP1 in all groups at day 7 (X200). Bottom: Representative renal sections of Masson’s trichrome staining in all groups at day 21 (X200). B: *Renal tissue TGF*β *levels*. Columns represent tissue TGFβ levels after 7 days from ureteral obstruction in all groups. Data are means ± SD. *p<0.001 vs sham, ^p<0.05 vs Vehicle D7. C: *FSP1 positive cells*. Columns represent FSP1 positive cells/HPF (means ± SD) at day 21. *p<0.001 vs all groups, °p<0.005 vs vehicle D21. D: *Interstitial fibrosis score* Columns represents the score of interstitial fibrosis (means ± SD) at day 21. *p<0.05 vs sham, °p<0.001 vs vehicle D21.

After 7 days of UUO, tissue TGFβ levels increased in vehicle group. In all treated groups, TGFβ levels were significantly lower (p<0.05). (Fig 6, panel B)

### 3.10 Renal fibrosis

Fibrosis was defined both as FSP1 positive cells/HPF and Masson’s trichrome positive area/HPF (X400). After 21 days of obstruction FSP1 positive cells increased significantly in non treated animals (p<0.001). (Fig 6, panel C) A significant decrease of fibroblast amount was observed following ACEi, MSC and combination therapies (p<0.005). Masson’s trichrome staining showed that fibrosis score was significantly (p<0.001) less severe in rats treated both with MSC and ACEi+MSC compared to untreated rats at day 21. (Fig 6, panel D)

### 3.11 Renal Human antigen R (HuR) mRNA, RENmRNA expression and Renin levels at day 1

**Fig 7:**
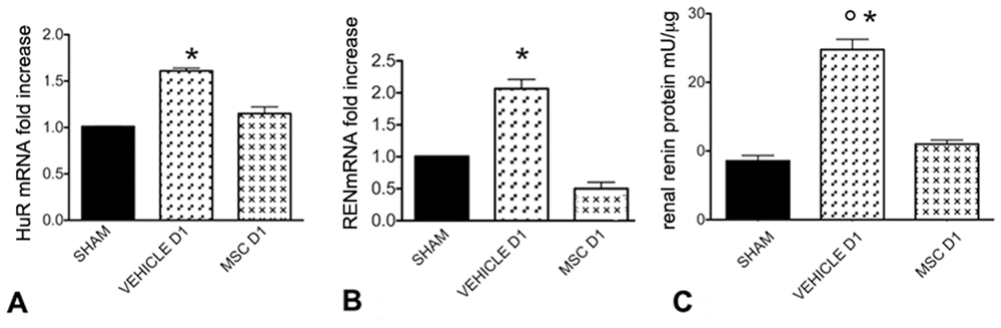
HuRmRNA, RENmRNA expression and renal Renin levels. A: HuRmRNA expression in Sham, Vehicle and MSC groups one day after ureteral ligation determined by real time PCR. Columns indicate HuRmRNA expression normalized to the βactin expression and converted in fold change. *p<0.001 vs Sham and MSC D1. B: RENmRNA expression in Sham, Vehicle, and MSC groups one day after ureteral ligation determined by real time PCR. Columns indicate RENmRNA expression normalized to the βactin expression and converted in fold change. *p<0.001 vs Sham, MSC D1. C: Renal Renin levels in Sham, Vehicle and MSC groups one day after ureteral ligation. Columns represent Renin levels. Data are means ± SD. °p<0.005 vs MSC D1, *p<0.0001 vs sham.

HuRmRNA, RENmRNA expression and Renin levels increased significantly 1 day after ureteral ligation in non treated animals (p<0.001). MSC treatment inhibited HuRmRNA, RENmRNA expression and Renin levels. (Fig 7)

### 3.12 *In vitro* experiments

#### 3.12.1 MSC/HK2 co-coltures

**Fig 8:**
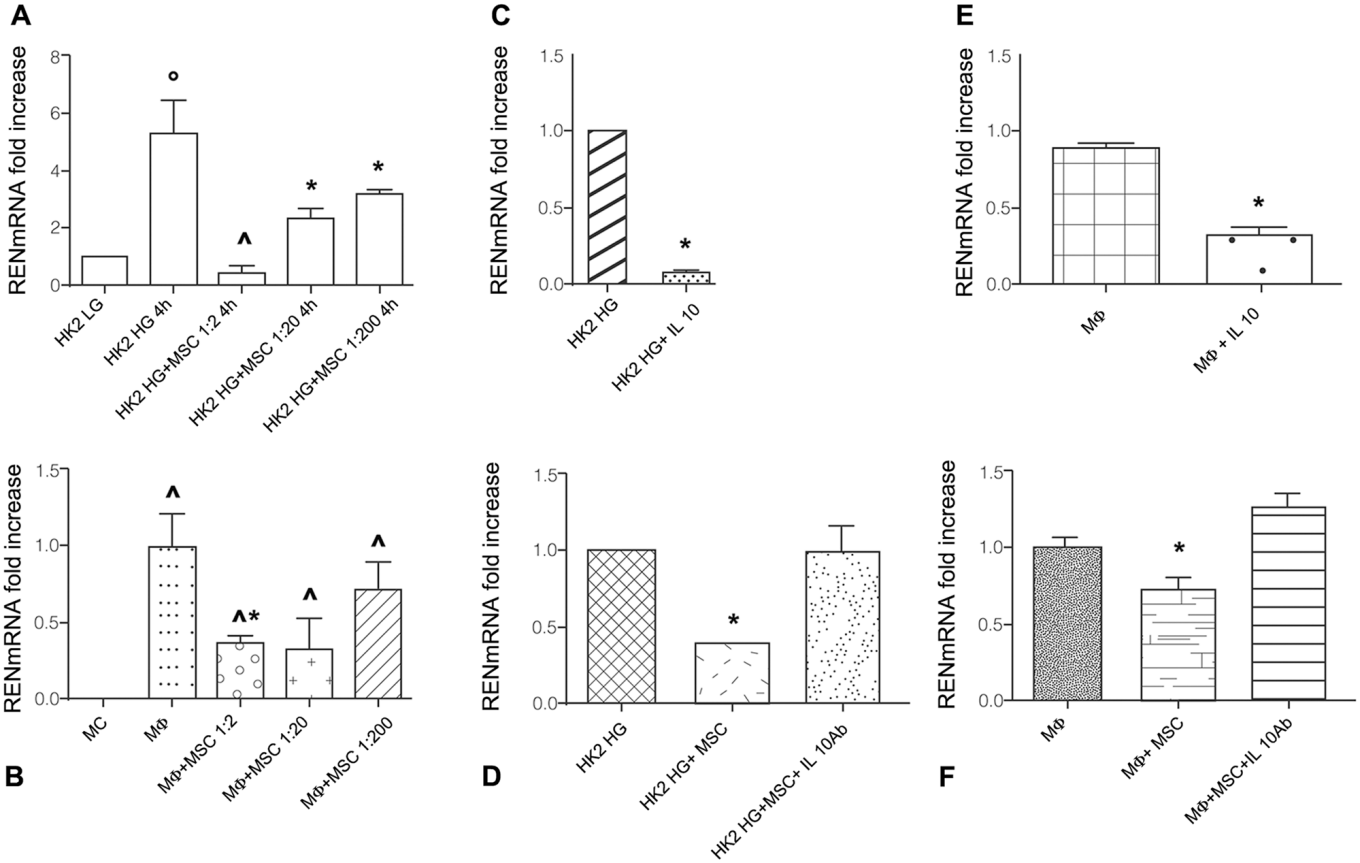
RENmRNA expression in co-coltures of MSC and HK2 or Mϕ. A: RENmRNA expression in HK2 cultured in low glucose (LG) medium, high glucose (HG) medium, in co-coltures with MSC (1:2, 1:20, 1:200) for 4 h. Columns indicate RENmRNA expression normalized to the βactin expression and converted in fold change. °p<0.05 vs HK2 LG; HK2 HG + MSC 1:2 4h. ^p< 0.01 vs HK2 HG + MSC 1:20 4h, HK2 HG + MSC 1:200 4h. *p<0.01 vs HK2 LG. B: RENmRNA expression in circulating monocytes (MC), macrophages (Mϕ), MSC+Mϕ co-coltures (1:2, 1:20, 1:200) for 4 days. Columns indicate RENmRNA expression normalized to βactin expression and converted in fold change. ^p<0.01 vs MC, *p<0.005 vs Mϕ. C: RENmRNA expression in HK2 cultured in HG medium in presence and absence of IL10 (10 ng/ml) for 1 h. *p<0.001 vs HK2 HG. D: RENmRNA expression in HK2 cultured in HG medium, in MSC/HK2 HG co-coltures (1:2) in presence and absence of anti IL10 blocking antibody at concentration 100 ng/ml for 4 h. Columns indicate RENmRNA expression normalized to the βactin expression and converted in fold change. *p<0.0001 vs HK2 HG, HK2 HG+MSC+anti IL10 Ab. E: RENmRNA expression in Mϕ cultured in presence and absence of IL10 (10 ng/ml) for 4 h. Columns indicate REN mRNA expression normalized to βactin expression and converted in fold change. *p<0.05 vs Mϕ. F: RENmRNA expression in Mϕ, MSC/Mϕ co-coltures (1:2) in presence and absence of anti IL10 blocking antibody at concentration 100 ng/ml for 24 h. Columns indicate RENmRNA expression normalized to βactin expression and converted in fold change. *p<0.05 vs Mϕ and Mϕ+MSC+IL10 Ab.

RENmRNA expression, as expected, was significantly higher in HK2 cultured in high glucose (HG) medium than in low glucose (LG) medium (p<0.05). (Fig 8, panel A) Presence of MSC in HK2 HG cultures reduced RENmRNA expression in a dose dependent manner. At 1:2 MSC/HK2 ratio RENmRNA expression was similar to HK2 LG expression. (Fig 8, panel A)

#### 3.12.2 MSC/Macrophages co-coltures

**Fig 9:**
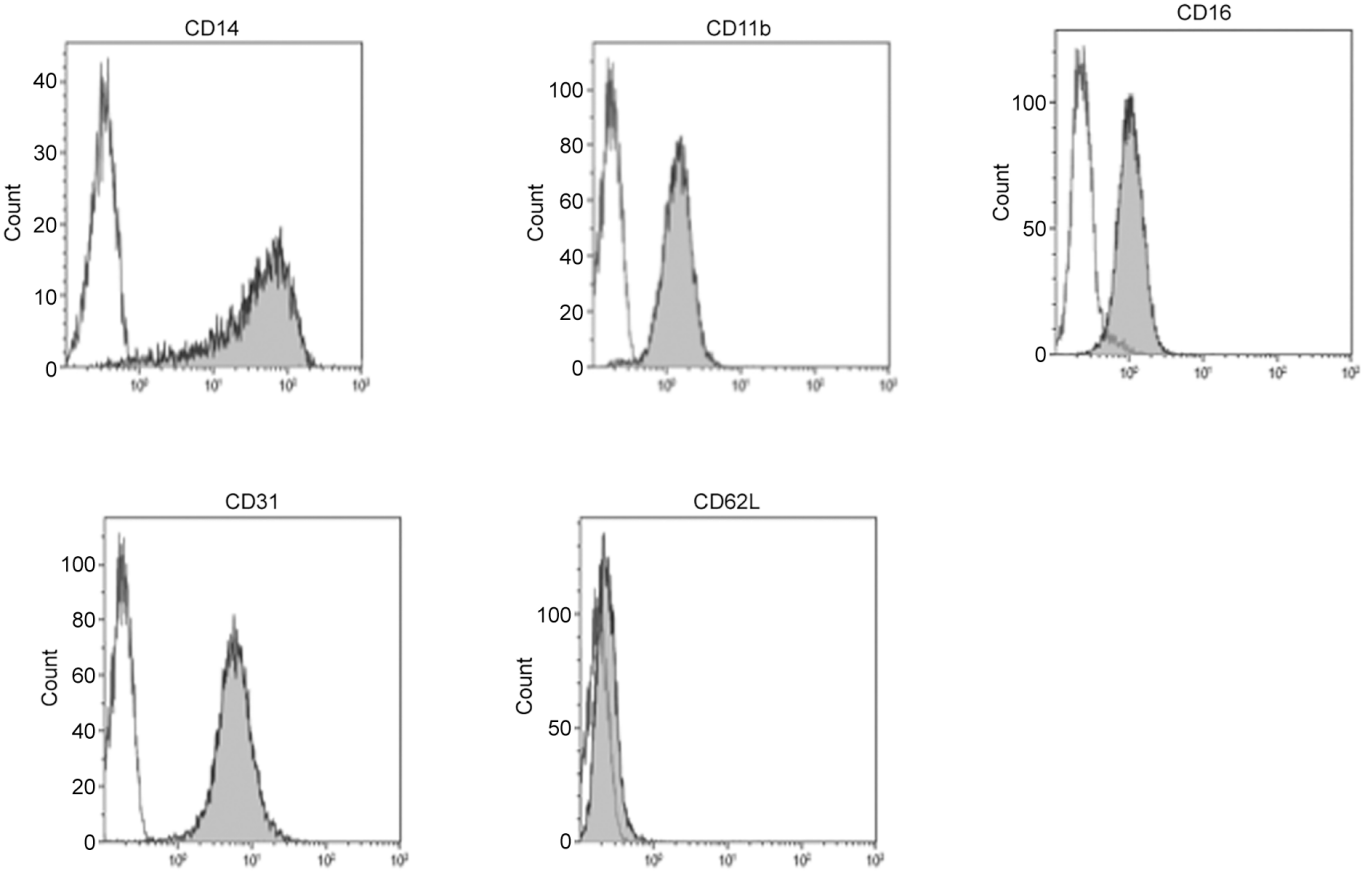
Representative flow cytometry analysis of monocyte-derived macrophages. Macrophages were obtained after 24 hours of adhesion. White peaks represent negative control by isotype-matched, non-reactive fluorochrome-conjugated antibodies. Grey peaks represent positive cells.

Macrophage characterization after 24 h adhesion is shown in Fig 9. After 4 days MSC/Macrophages co-colture, flow cytometry analysis revealed a significant increase of cell surface CD14 expression: Mean Fluorescence Intensity (MFI) before adhesion 46, MFI post adhesion 108. RENmRNA expression in Mϕ was significantly higher than in circulating MC (p<0.01).

MSC in co-culture reduced RENmRNA expression in Mϕ, significantly at 1:2 ratio (p<0.005). (Fig 8, panel B)

#### 3.12.3 MSC modulate RENmRNA expression via IL10

In order to investigate the mechanism through which MSC suppress RENmRNA expression, we performed *in vitro* culture experiments in presence of IL10 and anti-IL10 blocking antibody. Addition of IL10 to HK2 HG cultures significantly decreased RENmRNA expression (p<0.001). (Fig 8, panel C) While addition of anti-IL10 blocking antibody in MSC/HK2 HG co-culture reversed MSC suppression on RENmRNA expression. (Fig 8, panel D) Similarly RENmRNA expression in Mϕ cultures additioned with IL10 was significantly lower than in Mϕ without IL10 (p<0.05). (Fig 8, panel E) The addition of anti IL10 blocking antibody in co-cultures MSC/Mϕ reversed MSC suppressive effect. (Fig 8, panel F)

## Discussion

It is well documented that acute UUO is followed by a prompt renal vasodilatation that turns into a persistent vasoconstriction whithin 24 hours.[4–6] The ensuing ischemia is associated with the progressive development of tubular atrophy and interstitial fibrosis that are a proved effect of increased AII release resulting from enhanced Renin production.[2,3] In addition, recent studies have shown that Renin has a fibrogenic effect that is independent of its enzymatic release of AII. In fact, the interaction of Renin with its receptor activates the production of TGFβ transduced by the ERK system. [14–16] MSC have been shown to have an antiiflammatory and antifibrogenic effect in several models of injury to various tissues, including the kidney.[35–37,46] In the present study we have investigated whether MSC prevent UUO induced fibrosis and we have tested whether MSC operate this effect through Renin. Furthermore, we have compared the results obtained following MSC infusion with those of ACEi treatment and the combination of both in order to add therapeutic advantage. Our results show that MSC decrease tubular cell apoptosis and infiltrating monocyte number and preserve tubular integrity preventing luminal dilatation and disruption of the basement membrane. These effects were associated with a significant decrease of fibrotic renal tissue, that did not result in a significant effect on renal function, as indicated by serum creatinine levels. These findings may be argued as contradictory, but a sound explanation is provided by simply considering that the loss of renal function associated with fibrosis in the obstructed kidney is compensated by increased GFR of the contralateral organ.[47] Of course, prevention of fibrosis as operated by MSC or ACEi treatment limits the loss of function in the obstructed kidney, but does not induce any compensatory increase in controlateral function.

Kidney protection afforded by MSC was comparable to that provided by ACEi, while a further decrease of renal inflammation and interstitial fibrosis was observed with the combined use of MSC and ACEi. Our results provide more informations on the MSC therapeutic potential described in previous studies, where it has been shown that MSC injected into obstructed kidney reduce the expression of collagen synthesis, fibroblast activation [42] and infiltrating macrophage number.[37–41,43,48,49] However the main innovation of our study was to shed some light on the possible mechanism(s) that account for MSC effects. It is well known that in acute and chronic models, MSC may act by paracrine action rather than a transdifferentiation into renal resident cells. This hypothesis is supported by our finding that very few EGFP-MSC were tracked in the kidney.

Moreover, we observed that after UUO, MSC inhibited expression of RENmRNA, Renin, AII and aldosterone, suggesting that MSC may antagonize RAS. It has been shown that RAS activation induces recruitment of inflammatory cells in several disease models including UUO [7,8], with release of inflammatory, pro-apoptotic and fibrogenic cytokines that enhance tissue damage.[10,50–53] Therefore, RAS inhibition observed in MSC treated rats may also explain prevention of macrophage infiltration.

We also found that MSC abated TGFβ that plays a crucial role in generating fibrosis, because it stimulates matrix proteins synthesis, decreases matrix degradation, stimulates fibroblast proliferation and synthesis of stress fibres, promotes epithelial–mesenchymal transition of tubular cells.[11–13] This finding may be a consequence of Renin inhibition by MSC. A question raised by our study is how MSC affect Renin release. In renal tissue, granular cells of afferent arteriole are the main source of Renin, but RENmRNA is expressed in tubular cells [54,55] and infiltrating macrophages [56], too.

Since MSC prevent macrophage infiltration [44,48,49], it is reasonable to assume that one mechanism by which MSC inhibited RAS was by blocking the source of Renin represented by macrophages.[57] However, the behaviour of RENmRNA expression in rats treated with ACEi and MSC suggests that MSC directly downregulated Renin production by renal cells. We have shown that the increase in RENmRNA and Renin that occurs in ACEi treated rats is suppressed by MSC. This suppression takes place while the inhibition of AII caused by ACEi persists, so that MSC suppress Renin by a mechanism that does not involve the AII-evoked feedback. The evidence that MSC and ACEi operate RAS inhibition in a different way provides a rationale for using them in combined therapy. Actually in our study their combination strength their anti-fibrogenic effect.

In order to understand MSC mechanism, we focused on: a) Renin release, first step of RAS activation, b) HuR expression, that sustains Renin production, stabilizing RENmRNA [17–19,58,59] c) IL10, cytokine known to be produced by MSC [54–56,60,61] and able to inhibit HuR expression.[17–19]

For the first time we proved in *ex vivo* experiments that MSC reduce REN and HuR mRNA expression in obstructed kidney, already one day after UUO. To understand whether MSC inhibited REN and HuR mRNA expression via IL10, we performed *in vitro* experiments and demonstrated that MSC downregulated RENmRNA in tubular cells [62,63] and in macrophages[57]. IL10 inhibited RENmRNA expression in activated tubular cells and macrophages and the addition of anti IL10 blocking antibody in co-cultures of MSC/tubular cells and MSC/macrophages reversed MSC effects. We also measured IL10 in MSC culture surnatants but we found only slightly detectable levels (data not shown), perhaps due to the fast use of IL10 by the cells.

In conclusion, we have shown that MSC injection in UUO prevents inflammation, tubular cell apoptosis and fibrosis. These effects are associated with the suppression of RAS, macrophage infiltration and TGFβ expression. These findings allow to reconstruct a rational sequence of events that may represent a RAS inhibitory pathway driven by MSC. We confirm that RAS inhibition is associated with HuR suppression, and give evidence that MSC suppress HuR and thus inhibit RAS. IL10 released by MSC may be responsible for HuR suppression.

The present study is a further demonstration that MSC mitigate injury and/or favour repair by delivering soluble factors that in a paracrine or even endocrine manner modify the cell behaviour favouring kidney recovery.

## Abbreviations

MSC: Mesenchymal Stromal Cells
UUO: Unilateral Ureteral Obstruction
AII: Angiotensin II
RAS: Renin Angiotensin System
RENmRNA: Renin mRNA
TNFα: Tumour Necrosis Factor alfa
TGFβ: Transforming Growth Factor beta
HuR: Human Antigen R
ERK: extracellular-signal-regulated kinase
ACEi: Angiotensin Converting Enzyme inhibitor
SD: Sprague Dawley
EGFP: Enhanced Green Fluorescent Protein
P: Passage
PE: Phycoerithrin
HPF: High Power Field (x 400)
FSP1: Fibroblast Specific Protein 1
TBM: Tubular Basement Membrane
MFI: Mean Fluorescence Intensity
MC: monocytes
Mϕ: macrophages
